# Knowledge and Attitudes Regarding a Weight-Neutral Approach to Health Promotion in Primary Care Settings

**DOI:** 10.7759/cureus.83632

**Published:** 2025-05-07

**Authors:** Emily Ross

**Affiliations:** 1 Hospital Medicine, Aberdeen Royal Infirmary, Aberdeen, GBR

**Keywords:** cardiorespiratory fitness, health promotion, obesity, physical activity, primary care, weight loss, weight neutrality

## Abstract

Rising rates of obesity have caused much concern in recent years. However, research has consistently proven that when other variables are controlled, increased body size is not associated with morbidity or mortality and that intentional weight loss causes more harm than good. In response to this, many have urged a weight-neutral approach to health promotion. This would focus on increasing physical activity and cardiorespiratory fitness, metrics that do impact morbidity and mortality, regardless of change in body weight.

This study used a survey to discover what primary care clinicians know about physical activity and obesity with regard to health promotion and if their practice is in line with current guidelines. It found that the majority still hold the popular belief that weight loss is necessary to improve the health of overweight and obese individuals, and that they continue to recommend weight loss to their patients.

This exploratory study identified that the knowledge and practice of general practitioners (GPs) in the National Health Service (NHS) Grampian are not in line with current research and guidance about weight loss and health. Further research is recommended to establish the wider applicability of these findings and to further investigate how these misconceptions influence practice.

## Introduction

The prevalence of obesity in the UK has been rising for the last five decades. In 1975, an estimated 9.4% of UK adults were obese (BMI≥30). By 1996, that number rose to 21.9% and by 2016 to 27.8% [1]. This phenomenon has been described by many as an "obesity epidemic" [2].

Conventional wisdom states that this "epidemic" should be combated with weight loss, and many have advocated for encouraging overweight and obese individuals to lose weight [3]. In response, the prevalence of weight loss attempts has increased each year; in 1997, just 39% of UK adults reported trying to lose weight, in 2013 that number rose to 47% of the general population and 76% of those with a BMI≥30 [4].

Despite this, obesity rates continue to increase. Much of this can be attributed to weight loss attempts rarely being successful, with some studies showing that up to two-thirds of dieters regain more weight than they lost [5].

This increase in both obesity and weight loss attempts has been explained by Gaesser et al. (2011) [6] as being due to weight being repeatedly lost and regained in what they characterise as a “weight loss futile cycle” [7]. In this cycle, dieters fail to reach or maintain their weight loss goal. They then regain weight, often overshooting their original weight, before attempting to lose weight again.

Attention has been brought to the dangers of “weight cycling” in recent years, with it being associated with a 45% higher risk of all-cause mortality [8] and a 36% higher risk of death from cardiovascular diseases, irrespective of BMI [9]. Repeated dieting and "weight cycling" have also been associated with an elevated risk of eating disorders, anxiety and depression [10].

Even when weight loss is successful and maintained, it is not beneficial. Intentional weight loss in healthy overweight and obese individuals has been associated with higher mortality compared to those who don’t intentionally lose weight [11]. This also applies to patients with “obesity related” co-morbidities, as neither intentional weight loss nor weight gain is associated with changes in all-cause mortality for obese and overweight patients with type 2 diabetes [12]. In 2013, the "Look AHEAD" trial [13] compared obese and overweight patients with type 2 diabetes who were randomised to an intervention of intentional weight loss with a control group. The intervention group showed significant successful weight loss. However, the study was stopped for futility as results showed no difference in the cardiovascular morbidity or mortality between the two groups.

Some studies have found that intentional weight loss interventions do decrease mortality [14]. However, these studies fail to account for the increased levels of physical activity (PA) and cardiorespiratory fitness (CRF) that are gained as a side-effect of weight loss programmes [15]. More recent studies that do account for change in CRF show that an increase in CRF is directly associated with a reduction in morbidity and mortality regardless of changes in BMI, body weight, or body fat percentage [6,16].

Studies that find a positive correlation between obesity and mortality [17] also fail to account for CRF [18]. It has been suggested that conditions we think of as “obesity related” - hypertension, dyslipidaemia, metabolic syndrome and type 2 diabetes - are actually attributable to a low level of CRF [7]. Other studies have shown that obesity is often identified as a predictor of poor health because it is confounded with other variables such as dieting, discrimination, stress and social class [19,20].

When fitness is taken into account, it is evident that CRF has a greater impact on longevity than BMI. The relative risk of all-cause mortality is twice as high for unfit individuals (the bottom 20-25% of individuals for age-adjusted CRF), regardless of BMI. Moreover, mortality risk for moderately fit individuals (the top 75-80% of individuals for age-adjusted CRF) is the same for normal weight, overweight and obese individuals [21].

This is encouraging news as it is advised that a moderate level of CRF can be achieved with just 150 minutes of moderate intensity physical activity per week [21,22]. This is the same level of physical activity currently recommended in the UK Chief Medical Officers' Physical Activity Guidelines [23].

This evidence supports the change to a “weight-neutral” approach for managing “obesity related” health conditions as proposed by Gaesser and Angadi (2021) [7]. This approach focuses on increasing physical activity and CRF. Therefore, avoiding the risks associated with the weight loss futile cycle by moving the focus away from intentional weight loss. This shift in focus also ensures that normal-weight unfit individuals are not being overlooked. Promoting physical activity to all individuals regardless of BMI will improve health outcomes and reduce the relative risk of mortality for all [24,23].

Aim of the study

It was hypothesised that primary care clinicians who lead consultations and therefore have the opportunity to provide lifestyle advice during those consultations are not up to date with research and guidance regarding physical activity, weight loss and cardiorespiratory fitness. This hypothesis was derived from anecdotal evidence of weight loss being promoted to overweight and obese patients above all other lifestyle interventions during primary care consultations, and cardiorespiratory fitness rarely being encouraged. In order to investigate this further, the following research question was designed: How does the knowledge and practice of primary healthcare clinicians in the National Health Service (NHS) Grampian compare to current research and guidance regarding physical activity, weight loss and cardiorespiratory fitness?

The following subtopics on which clinician knowledge would be assessed were identified: physical activity guidance for adults; the dangers of weight cycling; health outcomes for overweight and obese people; the benefits of cardiorespiratory fitness compared to weight loss; a weight-neutral approach to health promotion.

This exploratory study aims to assess knowledge of these topics among a small group of primary care clinicians in NHS Grampian. This will be achieved using a survey to assess clinicians’ knowledge on the topics listed above and ask them to self-report on their practice with regard to health promotion. The survey results will be used to identify areas, if any, in which clinicians lack knowledge.

It was expected that some knowledge gaps would be identified and that this would establish the need for further, stronger research into whether these findings are applicable to a wider range of clinicians. Research demonstrating the areas in which knowledge gaps exist can be used to guide education programmes for primary healthcare clinicians, which would improve clinical practice by ensuring patients receive evidence-based health promotion advice.

## Materials and methods

Survey design

Data for this study were gathered by asking primary healthcare clinicians to report on their own knowledge and practices using an online survey.

The survey was designed using Google Forms (Appendices A-E). It comprised seven questions and was designed to be completed in under 10 minutes to maximise responses. Responses were anonymised to encourage honest answers. The majority of questions were multiple-choice style questions to facilitate data interpretation.

Question 1 asked participants what their role was to ensure that data was only collected from primary healthcare clinicians.

Question 2 asked participants to identify the current physical activity recommendation for adults from a list of six possible options, with only one being correct. Question 3 then asked participants to identify “moderate intensity physical activities” from a list of eight activities, with three of them (brisk walking, cycling and shopping) being true examples as defined by the UK Chief Medical Officers' Physical Activity Guidelines [23]. These two questions were designed to show if clinicians were up to date with current guidelines for physical activity in adults, as this knowledge or lack of knowledge may inform their practice when providing lifestyle advice.

Question 4 then asked participants to report on their own practice using a 5-point Likert scale. This question was designed to investigate if clinicians’ practice was up to date with current guidelines and evidence. Participants were asked how often they recommend weight loss and use BMI or weight as a way to assess health, as these are common practices that have been shown not to be based on evidence [7,11]. Participants were also asked how often they recommend physical activity and enquire about patients’ physical activity level, as these are evidence-based methods of assessing and promoting health [6,21].

Question 5 asked participants to mark six statements as either “true” or “false”. This question was designed to determine whether clinicians still hold key common misconceptions about the topics of obesity, weight loss and cardiorespiratory fitness, or if their knowledge is up to date with current evidence. The statements used in this question are: 

(a) “In general, intentionally losing weight reduces morbidity and mortality in overweight and obese people.” This is false [11].

(b) In general, an increase in cardiorespiratory fitness reduces rates of morbidity and mortality.” This is true [6].

(c) “Weight loss attempts are unsuccessful more often than they are successful.” This is true [5].

(d) “Intentional weight loss in overweight and obese patients has no effect on their likelihood of having a heart attack or stroke.” This is true [13].

(e) “Overweight and obese individuals have a higher mortality risk than normal weight individuals with the same level of cardiorespiratory fitness.” This is false [21].

(f) “Repeated dieting and ‘weight cycling’ (losing and regaining weight) has been associated with an increased risk of death, eating disorders, anxiety and depression.” This is true [10].

Question 6 asked participants if they were aware of “Fitness vs fatness on all-cause mortality: A meta-analysis” [21]; “Obesity treatment: Weight loss versus increasing fitness and physical activity for reducing health risks” [7] or “a ‘weight-neutral’ approach for obesity management” [7]. This question was designed to discover if clinicians are aware of these pieces of research, which have been crucial in addressing common misconceptions about weight loss and health.

Finally, question 7 asked participants to describe how knowledge of relevant research stated in question 6 had influenced their practice; this question was answerable by free text, and each response was analysed individually.

The survey was pilot tested by a general practitioner who provided feedback on question design. The answers provided by this general practitioner (GP) were not included in the results analysed for this study.

Survey distribution

The survey was distributed via email to the practice managers of 10 GP practices within NHS Grampian. As this is an exploratory study, these practices were chosen for convenience based on which practices were easily accessible and willing to participate. Each practice manager was asked to share the survey via email with staff who provide health promotion. They were advised that this included any members of staff who lead consultations during which lifestyle advice might be offered, including GPs, nurse practitioners, practice nurses, physician associates and allied health professionals. Each practice was also sent a poster containing a QR code which opened the survey when scanned and could be filled out on a mobile phone; the practices were advised to print and display this in a staff room or other relevant area. Survey respondents participated voluntarily, and no incentive was offered for participation. No minimum response target was set due to time constraints; all responses received during a two-week time period were analysed.

## Results

The survey received 27 responses, one of which had to be discounted as the participant reported their role as "Receptionist", and this study is assessing clinicians. The summary of clinicians who participated in the survey is represented in Table 1.

**Table 1:** Roles of survey participants

Role	Number of responses
General practitioner (GP)	18
Advanced nurse practitioner (ANP)	2
Nurse consultant	1
Practice nurse	4
Physician associate	1
Total	26

**Figure 1:**
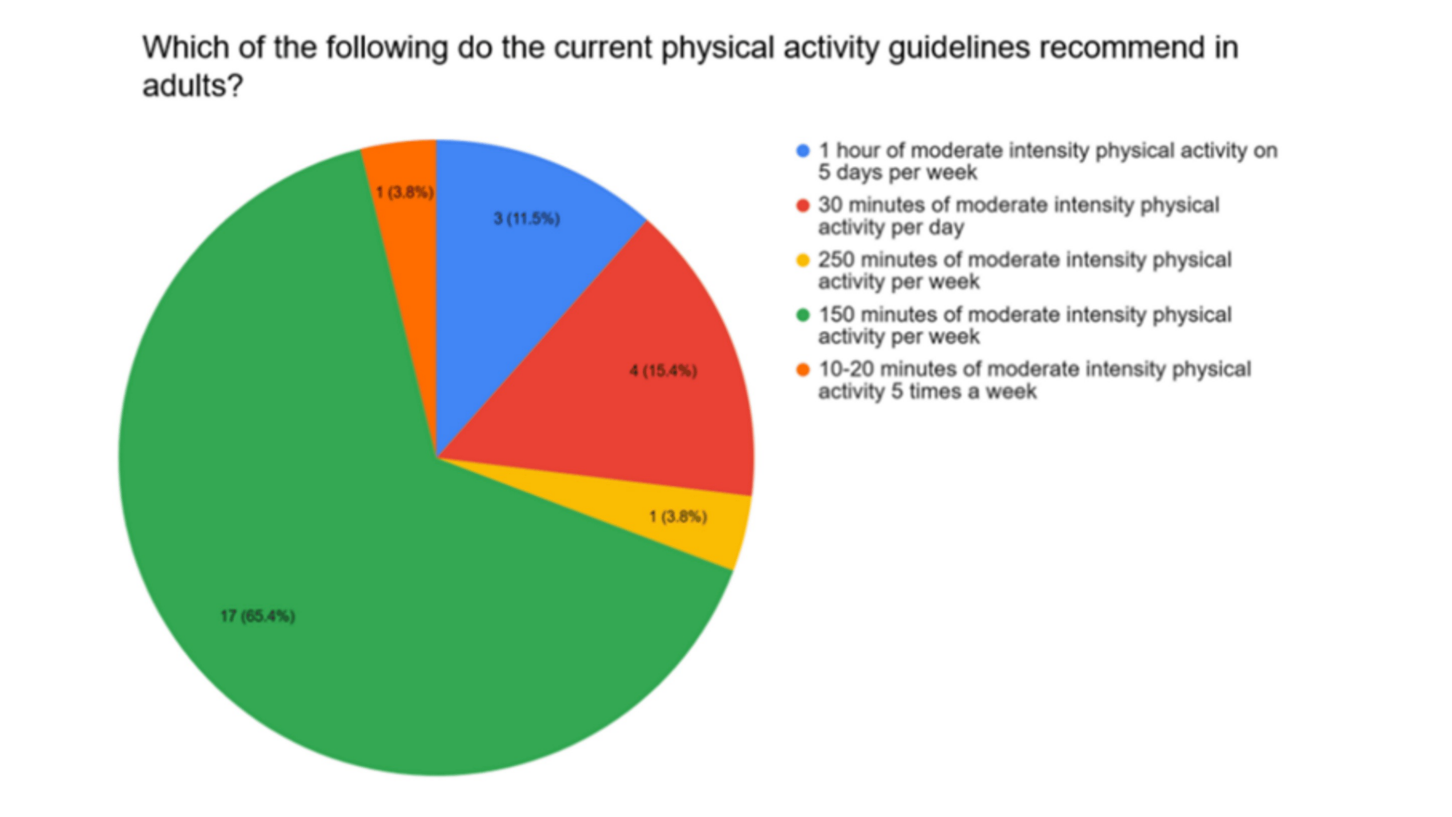
Current physical activity guidelines in adults

A total of 65.4% of participants correctly identified the current recommendation for physical activity in adults from six options (Figure 1).

**Figure 2:**
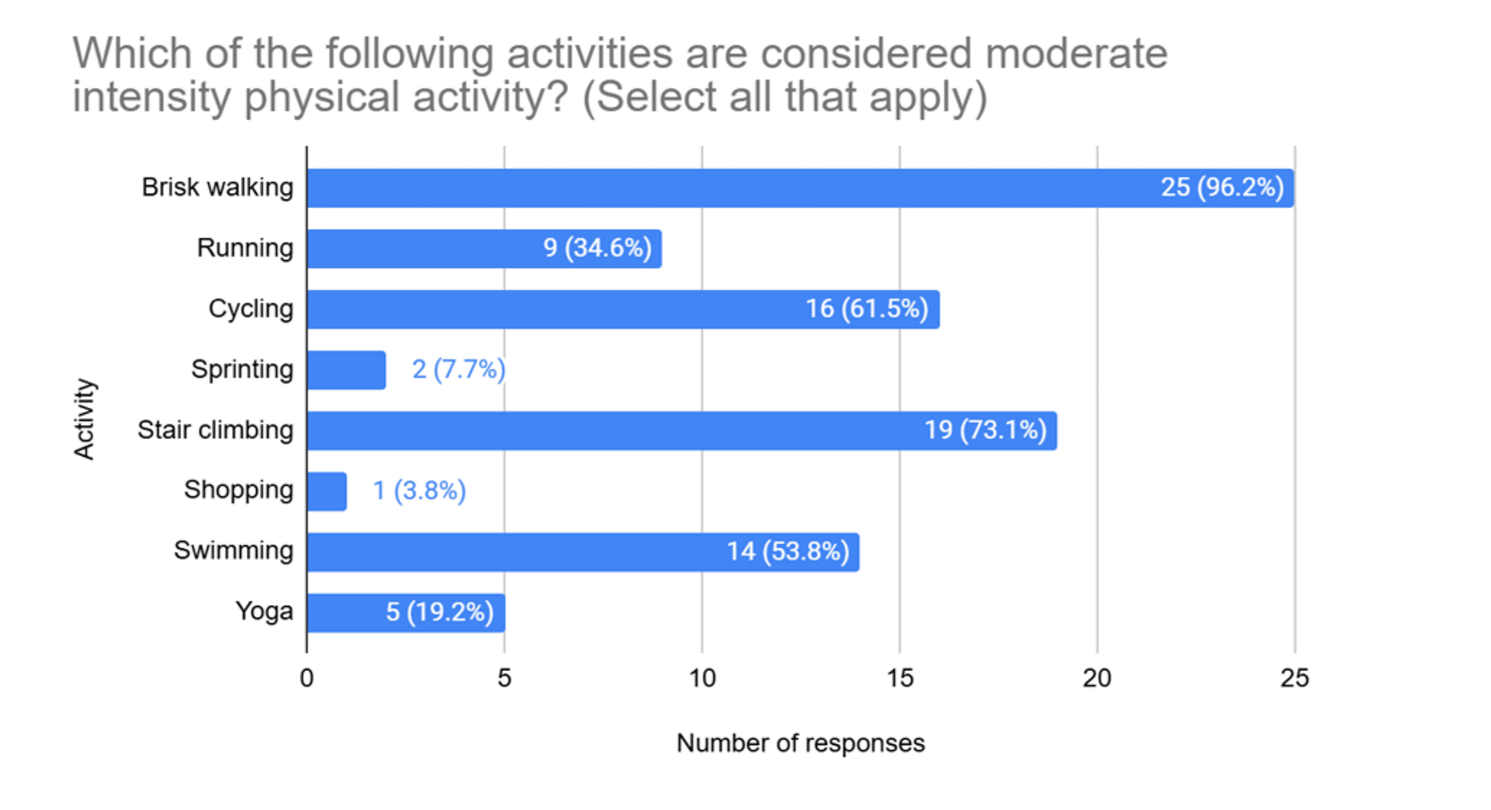
Forms of moderate intensity physical activity

When asked to identify examples of moderate intensity physical activity, 96.2% of participants correctly identified brisk walking as belonging to this category. A total of 61.5% identified cycling, and only one participant identified shopping (Figure 2).

**Figure 3:**
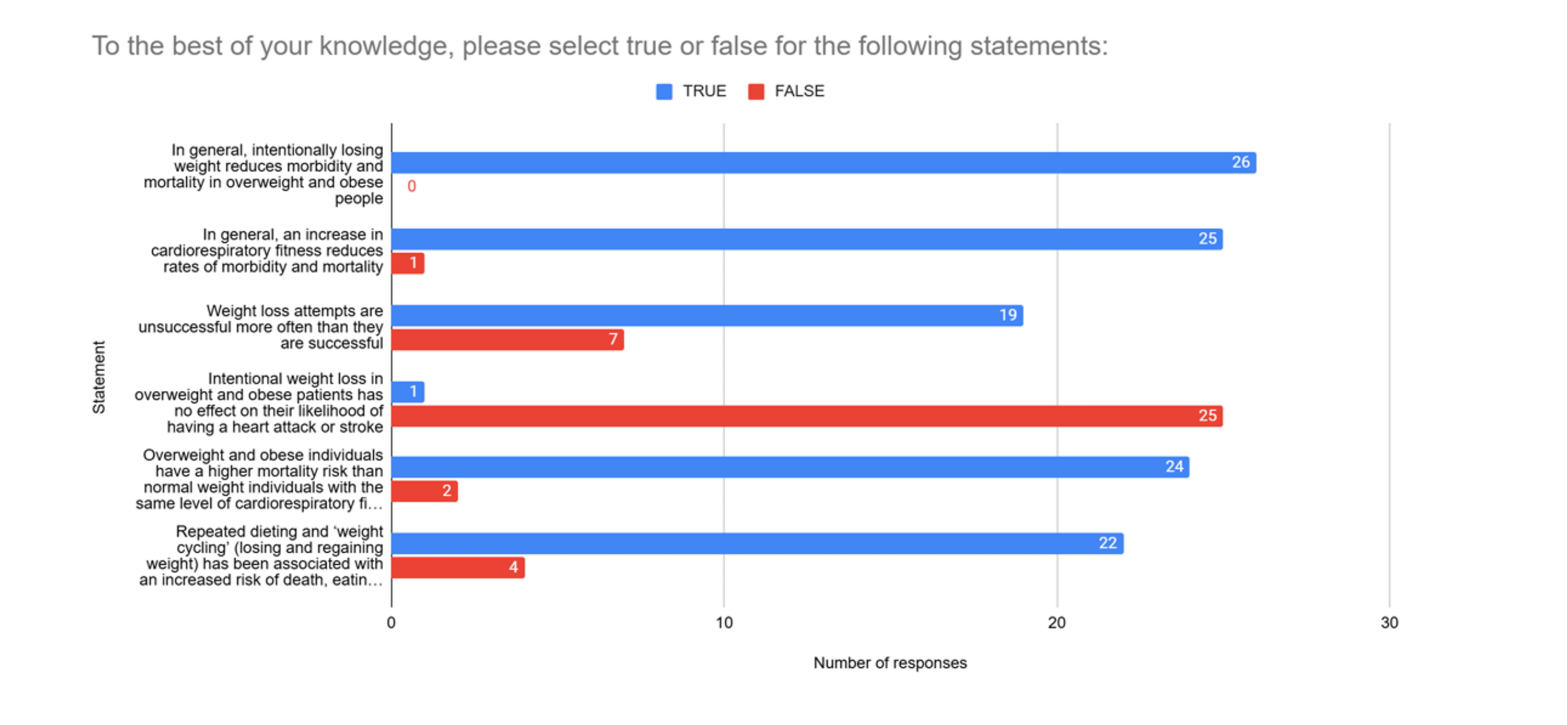
True or false statements

When responding to true or false statements (Figure 3), 100% of survey participants incorrectly reported that intentionally losing weight reduces morbidity and mortality in overweight and obese people.

Only 3.8% of participants were aware that intentional weight loss has been proven to have no effect on the incidence of heart attack and stroke in overweight and obese patients. 

A majority of participants (92.3%) incorrectly stated that overweight and obese individuals have a higher mortality risk than normal weight individuals with the same level of cardiorespiratory fitness. Additionally, 96.2% correctly identified that an increase in cardiorespiratory fitness reduces rates of morbidity and mortality. Furthermore, 73.1% of participants knew that weight loss attempts are more often unsuccessful than they are successful, and 84.6% of participants knew it to be true that repeated “weight cycling” has been associated with an increased risk of death, eating disorders, anxiety and depression.

**Figure 4:**
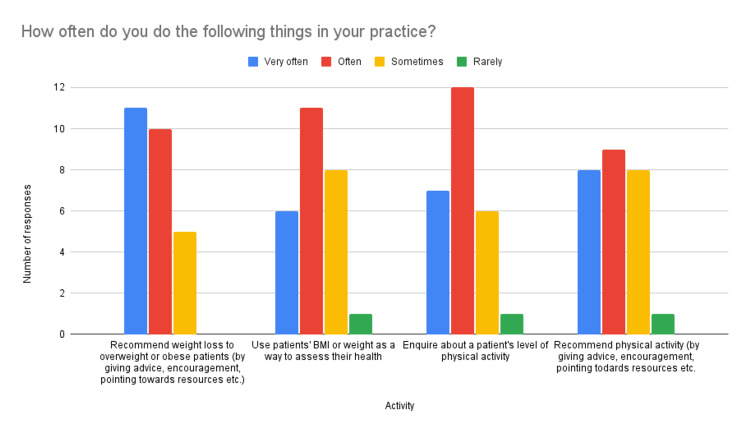
Reporting on own practice

Regarding participants’ own practice (Figure 4), participants recommend weight loss to patients more often than they recommend physical activity (42.3% very often, 38.5% often and 19.2% sometimes compared to 30.8% very often, 34.6% often, 30.8% sometimes and 3.8% rarely). However, when assessing patients’ health, they enquire about physical activity levels more often than they use BMI or weight (26.9% very often, 46.2% often, 23.1% sometimes and 3.8% rarely compared to 23.1% very often, 42.3% often, 30.8% sometimes and 3.8% rarely).

**Figure 5:**
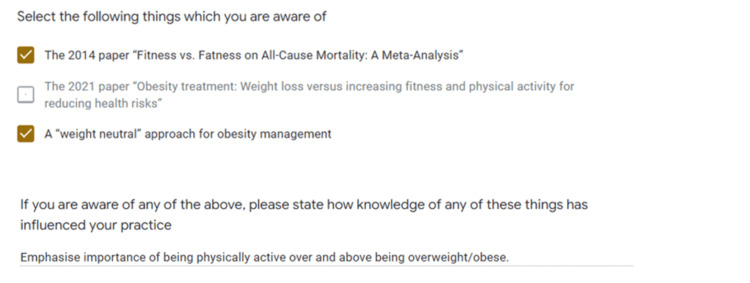
Awareness and influence of research

When asked about knowledge of current research (Figure 5), one participant selected that they were aware of the 2014 paper “Fitness vs. Fatness on All-Cause Mortality: A Meta-Analysis” and “A Weight-Neutral Approach for Obesity Management". Zero participants reported being aware of the 2021 paper “Obesity Treatment: Weight Loss Versus Increasing Fitness and Physical Activity for Reducing Health Risks”. When asked how knowledge of the above influenced their practice, this participant responded with “emphasise importance of being physically active over and above being overweight/obese."

## Discussion

Although most clinicians know that current physical activity guidelines recommend 150 minutes of moderate intensity physical activity per week, they tend to overestimate the intensity of “moderate intensity” physical activities. The UK Chief Medical Officers' Physical Activity Guidelines [23] give brisk walking, cycling and shopping as examples of moderate intensity physical activity. Although most survey participants identified brisk walking as belonging to this category, they were more likely to choose stair climbing (a "very vigorous" physical activity) over cycling, and only one participant correctly identified shopping as a form of moderate intensity physical activity. This means clinicians are likely advising more vigorous activity than is required to meet the recommendations. This may be daunting for many patients and discourage them from attempting to meet the guidelines if the recommendations seem unattainable.

Clinicians enquire about physical activity levels more often than they use BMI to assess health. This is a positive finding and may indicate that clinicians are aware of the importance of physical activity as a predictor of health. Likewise, the vast majority of clinicians knew it to be true that an increase in cardiorespiratory fitness reduces rates of morbidity and mortality. Conversely, in their own practice, clinicians recommend physical activity less often than they recommend weight loss, and 34.6% of clinicians reported recommending physical activity only sometimes or rarely. It is possible that although clinicians know the benefits of physical activity, they are unused to advising it and that this is an area requiring education.

The majority of clinicians providing health promotion in primary care understand that weight loss attempts are rarely successful and are aware of risks associated with "weight cycling" such as an increased risk of death, eating disorders, anxiety and depression [8-10]. Despite this, most clinicians continue to frequently recommend weight loss to overweight and obese patients and do so more often than they recommend physical activity. Furthermore, more than half of clinicians admit to using BMI or weight as an indicator of patient health, often or very often. This is likely due to the fact that, according to this study, 100% of clinicians surveyed hold the widespread misconception that intentional weight loss improves health outcomes for obese and overweight individuals, and the vast majority believe that obesity is directly associated with mortality.

This misconception has the potential to cause harm. The idea of "healthism" - that individuals can have control over their health by adhering to certain behaviours [25] - has led to the medicalisation of body size [20]. Many clinicians believe that obesity itself is a disease that must be treated [26]. The problem with this view is that repeated instructions to lose weight lead to a perception that clinicians do not take the problems of overweight and obese patients seriously and attribute all symptoms to being due to weight [20]. There is likely some truth in this; a 2006 study found that obese patients were 1.65 times more likely than non-obese patients to have significant undiagnosed medical conditions found on autopsy [27]. This perceived weight discrimination leads to avoidance and delay in seeking healthcare, which has been linked to higher morbidity and mortality even when controlled for smoking, physical activity and disease burden [28]. Pressure to lose weight also causes increased body weight dissatisfaction, which is associated with increased rates of type 2 diabetes, cancer, hypercholesterolaemia and hypertension (as well as poorer health behaviours such as lower physical activity level and higher alcohol consumption) regardless of BMI [29,30]. Constant recommendations to lose weight may be especially harmful to those patients who are trying but unable to lose weight due to being stuck in the weight loss futile cycle [6].

All the above evidence supports a complete shift from a weight-centric to a weight-neutral approach to health promotion [7]. Not only is there evidence that intentional weight loss, even when successful, doesn’t improve morbidity or mortality, but it has been proven that a constant emphasis on weight loss from clinicians causes measurable harm to overweight and obese patients by causing body weight dissatisfaction and avoidance of healthcare.

Strengths

The survey was distributed to 10 GP practices throughout NHS Grampian, meaning data had the potential to be collected from a range of practices and likely does not reflect the culture of one specific workplace. The survey was hosted on an online platform, which provided anonymity, making the data collected more reliable.

Limitations

This study relied entirely on clinicians' self-reporting on their own practice and may not accurately reflect their true practice. The small number of survey respondents makes it unclear if these findings are generalisable to a larger population of primary care clinicians. Furthermore, the study was limited in geographic scope and in the variety of primary healthcare clinicians represented. A total of 69.2% were GPs, and all were working in NHS Grampian. This, therefore, limits the conclusions that can be drawn about other primary care professionals and clinicians in the rest of the UK.

Lastly, as the survey was distributed by practice managers rather than researchers, it is unknown how many clinicians received the survey and if it was distributed as advised. This makes it impossible to calculate a response rate or assess potential selection bias. Finally, despite the survey being pilot tested before use, it was not formally validated.

## Conclusions

GPs in Grampian are not up to date with current research and guidance about weight loss and health, and continue to hold misconceptions around these topics. When delivering health promotion, they report promoting weight loss over physical activity and fitness. This study is limited by its small sample size, limited geographic scope and relative lack of representation of clinicians other than GPs. It also relies entirely on self-reported practice with no objective evidence of behaviour. Further research is required to establish whether these findings are applicable to a wider and more varied population of primary healthcare clinicians. Additional research should aim to gather observed behaviour of clinicians to expand upon the self-reported evidence gathered for this study and further investigate how misconceptions around weight loss and health influence practice.

Education on the weight-neutral approach to health promotion is recommended to address demonstrated knowledge gaps. Following this, comprehensive research will be conducted to investigate whether or not this education will lead to a change in clinical behaviour and patients receiving evidence-based advice.

## Figures and Tables

**Figure 6:**
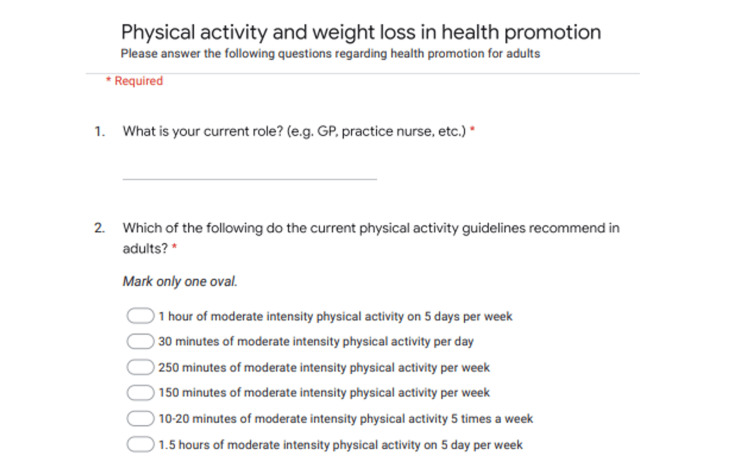
Google Form survey questions 1-2

**Figure 7:**
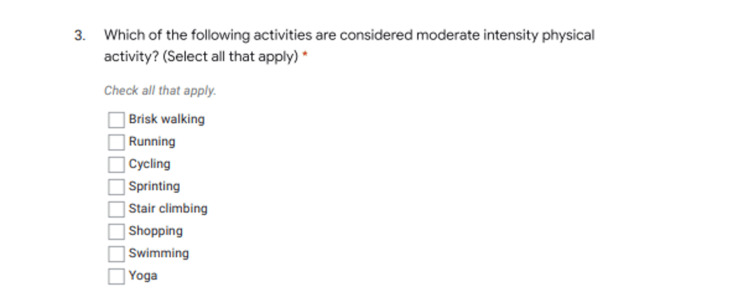
Google Form survey question 3

**Figure 8:**
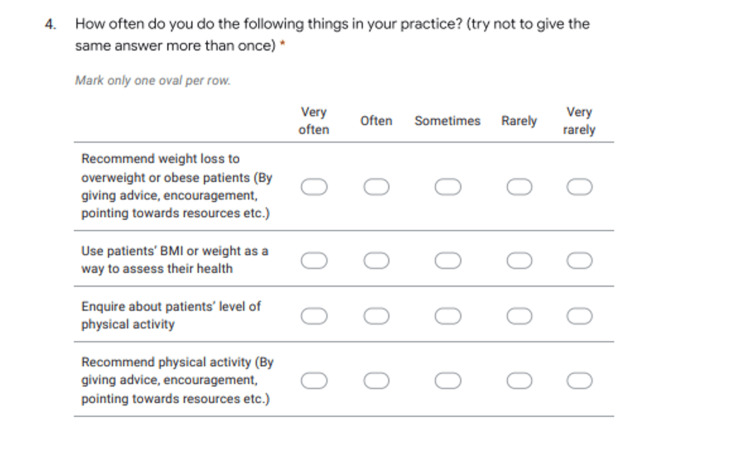
Google Form survey question 4

**Figure 9:**
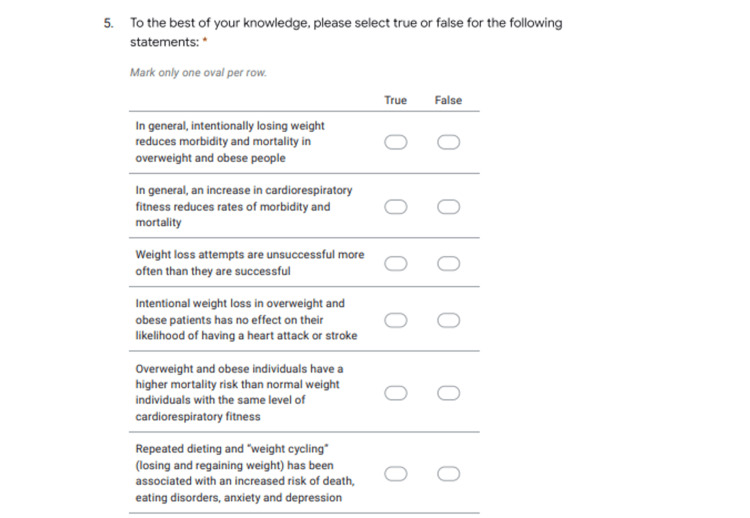
Google Form survey question 5

**Figure 10:**
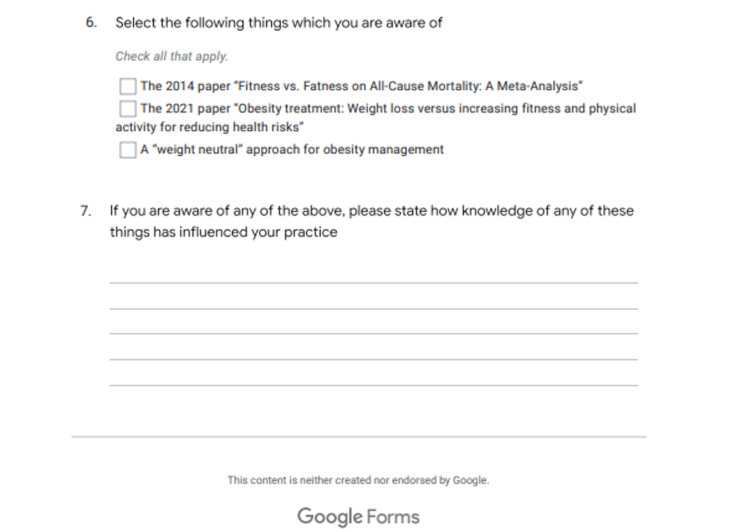
Google Form survey questions 6-7

## Appendices

Appendix A 

Appendix B 

Appendix C 

Appendix D 

Appendix E
